# Novel quinoxaline-based survivin degraders overcome docetaxel-resistance in castration-resistant prostate cancer

**DOI:** 10.1016/j.drup.2026.101356

**Published:** 2026-01-12

**Authors:** Caoqinglong Huang, Xunzhen Zheng, Qingbin Cui, Robert C. Peery, Zizheng Dong, Xiaohong Li, Jing-Yuan Liu, Jian-Ting Zhang

**Affiliations:** a Department of Cell and Cancer Biology, University of Toledo College of Medicine and Life Sciences, Toledo, OH 43614, USA; b Department of Medicine, University of Toledo College of Medicine and Life Sciences, Toledo, Ohio, USA

**Keywords:** Survivin dimerization, Degrader, Docetaxel resistance, Synergism, Prostate cancer

## Abstract

Survivin, a homodimeric protein in the Inhibitor of Apoptosis Protein (IAP) family, plays a dual role in apoptosis inhibition and cell cycle regulation. Overexpressed in many cancers but absent in most adult tissues, survivin is a compelling therapeutic target linked to disease progression, aggressiveness, and drug resistance. However, its structural properties render it “undruggable” by conventional approaches. Here, we present a transformative strategy to overcome this challenge by targeting survivin’s hydrophobic dimerization interface, inducing proteasome-dependent degradation. Building on the initial discovery of the survivin degrader LQZ-7I, we developed optimized analogs with significantly enhanced potency through medicinal chemistry. Our top-performing compounds, 7I10 and 7I14, selectively disrupt survivin dimerization, leading to its degradation and spontaneous apoptosis in castration-resistant prostate cancer (CRPC) cells. We also showed that survivin contributes to acquired resistance to docetaxel, the frontline chemotherapy for metastatic CRPC, and that the survivin degraders exhibit potent synergy with docetaxel, and the combination of 7I14 and docetaxel synergistically eliminates CRPC xenografts without added toxicity. This work introduces a first-in-class therapeutic approach that overcomes long-standing barriers to drugging survivin, offering a new avenue for combating docetaxel-resistant metastatic CRPC. With robust efficacy, a favorable safety profile, and potential for clinical translation, 7I10 and 7I14 represent significant advancements in the development of targeted cancer therapies to overcome docetaxel resistance.

## Introduction

Survivin (BIRC5) is the smallest member of the Inhibitor of Apoptosis Protein (IAP) family, with a single N-terminal Baculovirus Inhibitor of Apoptosis Protein Repeat (BIR) domain comprising a zinc-chelating motif, and a C-terminal alpha-helical coiled coil domain (Chantalat et al., 2000; Verdecia et al., 2000). This 16.5-kDa protein exists as a homodimer and presents a bowtie-shaped structure, centering around a hydrophobic dimerization interface enclosed by non-polar and aromatic residues (Chantalat et al., 2000; Verdecia et al., 2000). As an IAP, survivin inhibits both intrinsic and extrinsic apoptosis pathways alone or in complexes with other IAPs (Dohi et al., 2004; Eckelman et al., 2008; Suzuki et al., 2000; Tamm et al., 1998). Survivin overexpression is common in most solid malignancies and leukemia, while differentiated adult tissues have rarely detectable levels of survivin protein (Ambrosini et al., 1997). Survivin expression also negatively associates with prognosis and survival in many cancers, and it contributes to resistance to a wide variety of therapeutics (Cohen et al., 2003; Kawasaki et al., 1998; Rödel et al., 2002; Tanaka et al., 2000). It has also been observed that inhibiting survivin by means of biologicals including ribozymes, small-interfering RNAs, microRNAs, dominant negative survivin and small molecule inhibitors all induce spontaneous apoptosis of various cancer cells and sensitize them to chemotherapeutics in preclinical studies (Albadari and Li, 2023; Chen et al., 2000; Cui et al., 2023; Li et al., 2019; Mirzaei et al., 2023; Olie et al., 2000; Pennati et al., 2004; Pennati et al., 2002; Tanzadehpanah et al., 2023). These observations collectively render survivin an ideal target for developing cancer therapeutics.

Despite decades of research, no true small molecule survivin inhibitor that directly binds to and inhibits survivin has moved into clinical testing. The slow progress mainly attributes to the fact that survivin is considered “undruggable” or difficult to inhibit because it lacks enzymatic activities or an active pocket for a small molecule inhibitor to bind to. Although different approaches targeting the upstream regulators of survivin expression including transcription factors have been tested, none of these strategies have been successful due to a variety of issues including indirect targeting of survivin expression and poor pharmacokinetics (Cui et al., 2023).

Recently, targeting the homodimerization domain of survivin to induce its proteasome-dependent degradation has led to the discovery of true and effective survivin inhibitors, LQZ-7F and LQZ-7I of different scaffolds, leading to survivin degradation (Peery et al., 2020; Qi et al., 2016). These survivin degraders do not need a ligand for E3 ligase, different from other known PROTAC. A recent medicinal chemistry study to improve the quinoxaline-based LQZ-7I resulted in several derivative compounds with significantly better cytotoxicity and improved potency (Cui et al., 2025). In this study, we conducted detailed biological evaluation of these LQZ-7I analogs and identified LQZ-7I10 (7I10) and LQZ-7I14 (7I14) as the best leads for further study. These leads not only effectively induced proteasome-dependent survivin degradation and spontaneous apoptosis by inhibiting survivin dimerization, but also synergized with docetaxel, a first-line chemotherapeutics for metastatic castration-resistant prostate cancer (CRPC), which normally ends with docetaxel resistance and increased risk of mortality for prostate cancer patients. These findings underlie the therapeutic potential of 7I10 or 7I14 as single agents and combinational therapy to overcome docetaxel resistance by directly targeting survivin protein for clinical evaluations.

## Materials and Methods

### Materials

Antibodies against survivin (#2808, 1:2000 dilution), cleaved caspase-3 Asp175 (#9661, 1:500 dilution), cleaved PARP Asp214 (#9541, 1:1000 dilution), PARP1 (#9532, 1:2000 dilution), IAP family antibody kit (#9770, 1:1000 dilution), and survivin siRNA (#6351) were purchased from Cell Signaling Technology^®^ (Danvers, MA). The antibodies against β-Actin (#A5316, 1:10000) and 14–3–3σ (#AF4424, 1:2000) were from Sigma-Aldrich^®^ (St. Louis, MO) and R&D Systems^®^ (Minneapolis, MN), respectively. HRP-conjugated secondary antibodies (#1721011, #1706515, and #sc-2354) were from Bio-Rad (Hercules, CA) or Santa Cruz Biotechnology (Dallas, TX). Scrambled control siRNA (#AM4611) and fetal bovine serum (FBS) (#10437–028) were from Invitrogen and GIBCO, part of the Life Science Technologies (Waltham, MA). Cell culture medium RPMI 1640 (#10–040-CV) and MEM (#10–010-CV) were from Corning^®^ (Corning, New York). All survivin-degrading compounds were synthesized in-house with quality and purity determined as described previously (Cui et al., 2025). The Halt^™^ protease and phosphatase inhibitor cocktail was purchased from Thermo Fisher (Waltham, MA). Other chemicals and reagents were purchased from Sigma-Aldrich^®^, Cayman Chemical (Ann Arbor, MI), VWR Chemicals (Radnor, PA), and Thermo Fisher.

### Cell lines and cell viability assay

Prostate cancer cell lines PC-3, C4–2, DU-145, 22Rv-1, and primary normal human dermal fibroblasts were from ATCC (Manassas, VA) and C4–2/Vector and C4–2/HA-Survivin clones were from a previous study (Peery et al., 2020), all have been authenticated as previously described (Peery et al., 2022; Peery et al., 2020). They were cultured in RPMI 1640 supplemented with 10 % (v/v) heat-inactivated fetal bovine serum (FBS). The docetaxel resistant DU-145^Doc^ was generated by stepwise selection of DU-145 cells with docetaxel at initial concentration of 0.1 nM, which is lower than its IC_50_, and gradual escalation of docetaxel to a final concentration of 100 nM. DU-145^Doc^ was maintained in the presence of 100 nM docetaxel. The primary normal human fibroblasts were cultured in serum-free fibroblast basal medium plus fibroblast growth kit from ATCC. The mouse MC3T3-E1 cell line was cultured in MEM Alpha supplemented with 10 % heat-inactivated FBS. All cells were maintained at 37°C with 5 % CO_2_ in a humidified incubator.

Cell viability was determined using methylene blue assay as we previously described (Peery et al., 2022; Peery et al., 2020). Briefly, cells were plated at a density of 2,000 cells/well in 96-well clear-bottom plates with 100 μL/well complete medium and cultured overnight, followed by treatments with survivin inhibitors for 72 h under the same condition. The cells were then fixed in ice-cold methanol overnight at −20°C and then stained with 100 μL/well 1 % methylene blue for 1 h. The cells were then washed three times with 10 mM borate buffer and allowed to air dry overnight before extraction of dye using 50 % (v/v) ethanol in 0.1 N HCl for determination of OD_650 nm_ using SpectraMax iD5a plate reader (Molecular Devices, San Jose, CA). IC_50_ was derived from survival curves using the GraphPad Prism 10 (Boston, MA). Relative resistance factor (RRF) was calculated using the equation RRF=IC_50(test)_/IC_50(control)_.

### Cell lysate preparation and Western blot analysis

Cell lysate preparation and Western blot analysis were performed as previously described (Peery et al., 2022; Peery et al., 2020). Briefly, cells were harvested for preparation of whole cell lysates in RIPA buffer supplemented with the Halt^™^ protease and phosphatase inhibitor cocktail. Protein concentrations were determined using the Bio-Rad Protein Assay kit (#5000006). The lysates were then subject to separation using SDS-PAGE and proteins were transferred to PVDF membranes, which were blocked in non-fat dry milk, probed with primary antibodies followed by HRP-conjugated secondary antibody. The signal was visualized using WesternBright Quantum HRP substrate (#K-12042, Advansta, San Jose, CA) and captured by X-ray film. Quantification of signals was performed using ImageJ (National Institutes of Health, Bethesda, MD).

### Cell lysis by perfluoro-octanoic acid (PFO) and Western blot analysis

PFO is an ionic detergent that maintains oligomerization state of proteins and has been used to lyse cells to determine the size of protein complexes by PFO-based polyacrylamide gel electrophoresis (PFO-PAGE). PFO-PAGE analysis of survivin was conducted similarly to a previous study (Xu et al., 2004). Briefly, 2 × 10^6^ cells were seeded overnight in 10 cm dishes followed by treatment with 0.6 ng/mL docetaxel for 3 days to enhance survivin level. The cells were then treated overnight with 10 μM MG132 together with DMSO vehicle, 2 μM 7I10, or 4 μM 7I14 before lysis using PFO lysis buffer (50 mM Tris/HCl, pH 8.3, 1 % PFO, 1 % NP40, 20 % glycerol, 0.005 % bromophenol blue, 1 mM ZnSO4, 15 mM 2-mercaptoethanol) supplemented with Halt^™^ protease and phosphatase inhibitor cocktail.

The cell lysate was digested with 3 units/μL micrococcal nuclease at 37 °C for 10 min and cleared of insoluble materials by centrifugation at 10,000 × g for 10 min. The supernatant was collected and proteins separated by PFO-PAGE using running buffer containing 25 mm Tris, pH 8.5, 192 mm glycine, and 0.1 % PFO as previously described (Xu et al., 2004). The proteins were then transferred to PVDF membranes for Western blot analysis probed using survivin antibody as described above.

Linear regression was used to determine the molecular weight of survivin from molecular weight standards separated by PFO-PAGE. The molecular weight of protein markers was used directly for plotting without logarithmic transformation as the markers are in the narrow range and better R^2^ was generated than using logarithmic transformation.

### RNA extraction and real-time RT-PCR

C4–2 or PC-3 cells were seeded at 3 × 10^6^ cells/plate in 10 cm dishes, treated with DMSO, 8 μM 7I10 or 7I14 for 6 h, followed by RNA extraction using an RNA PureLink^™^ RNA Mini Kit (#12183025, Invitrogen) according to manufacturer’s protocol. Reverse transcription was performed using a High-Capacity cDNA Reverse Transcription Kit (#4368814, Applied Biosystems^™^, Waltham, MA), followed by real-time PCR analysis using a SYBR Green qPCR Kit (#QS1005, Radiant^™^, Alkali Scientific, Fort Lauderdale, FL) on an Applied Biosystems^™^ 7500 Real-Time PCR system with the following primers for survivin: 5’-TGACGACCCCATAGAGGAAC-3’ (forward) and 5’-TGGCTCTTTCTCTGTCCAGTT-3’ (reverse), and for actin control: 5’-TGGCACCCAGCACAATGAA-3’ (forward) and 5’- CTAAGTCATAGTCCGCCTAGAAGCA-3’ (reverse).

### Mammalian two-hybrid assay

The mammalian two-hybrid assay was performed also as previously described (Peery et al., 2022; Peery et al., 2020) using the constructs generated in these studies. Briefly, PC-3 cells in 6-well plate at 0.8 × 10^6^ cells/well were transfected with pM-Survivin, pVP16-Survivin, pG5SEAP, and pRL Renilla in a ratio of 5:5:1:0.1 using Lipofectamine^™^ 3000 Reagent (#L3000015, Invitrogen). Empty vectors were used as negative controls in place of survivin-containing constructs. At 24 h post-transfection, cells were replenished with serum-free medium containing 2 μM 7I10 or 7I14 and incubated for 24 h. The conditioned media were then collected to measure the activity of secreted embryonic alkaline phosphatase (SEAP) reporter using Great EscAPe^™^ SEAP Chemiluminescence Kit 2.0 (#631737, Takara Bio, San Jose, CA) following the manufacturer’s protocol on Sirius FB12 Luminometer (Berthold Technologies, Bad Wildbad, Germany). Renilla luciferase activity from pRL Renilla (#E2241, Promega, Madison, WI) was also measured using the Renilla Luciferase Assay System (#E2810, Promega) according to manufacturer’s protocol and used as a transfection efficiency control.

### Cycloheximide chase assay

Cycloheximide chase assay was performed as previously described (Qi et al., 2016). Briefly, C4–2 or PC-3 cells were seeded at 1 × 10^6^ cells/plate in 10 cm dishes. At different times following treatment with DMSO vehicle control, or 2 μM 7I10 or 7I14, cycloheximide was added to a final concentration of 10 μM. Cells were then harvested for Western blot analysis of survivin and actin control. The survivin level was quantified for determination of survivin half-life (t_1/2_) using GraphPad Prism 10 (Boston, MA).

### Apoptosis assay

Cell death and apoptosis were determined using the Vybrant^™^ DyeCycle^™^ Violet/SYTOX^™^ AADvanced^™^ Apoptosis Kit (#A35135, Invitrogen), which contains a cell-permeant DyeCycle^™^ Violet dye that stains condensed chromatin of apoptotic cells and an impermeant SYTOX^™^ AADvanced^™^ dye that stains necrotic cells. Briefly, C4–2 and PC-3 cells were seeded in 10 cm dishes at 2 × 10^6^ cells/dish, treated with DMSO vehicle control or 4 μM 7I10 or 7I14 for 24 h, and harvested for staining using the kit according to manufacturer’s instructions. The stained cells were then analyzed using a BD FACSAria IIu High-Speed Cell Sorter (BD Biosciences, Franklin Lakes, NJ) with data analyzed using the FlowJo^™^ v10 software (BD Biosciences).

Caspase activity was determined using Caspase-Glo^®^ 3/7 Assay System (#G8091, Promega) according to manufacturer’s instructions. Briefly, 4–2 or PC-3 cells were seeded at 2000/well in 96-well plates, treated with DMSO vehicle control, 7I10 or 7I14 for 24 h, followed by caspase activity assay using the Caspase-Glo^®^ 3/7 Assay System.

### In-vivo efficacy and toxicity assay

All animal experiments were performed in adherence to the NIH Principles of Laboratory Animal Care and were approved by the Institutional Animal Care and Use Committee of the University of Toledo. For the *in-vivo* efficacy study, 3 × 10^6^ PC-3 cells in media were implanted in the hind flanks of 6-week-old NSG male mice. After the tumor volume reached approximately 60 mm^3^ in volume, the mice were randomized into four groups with 3 or 4 mice in each group. The mice were treated with vehicle (group 1), 15 mg/kg 7I14 (group 2), 5 mg/kg docetaxel (group 3), or 15 mg/kg 7I14 plus 5 mg/kg docetaxel (group 4). 7I14 was administered twice a week while docetaxel was given once a week via IP for a total 6 treatments. The bodyweight and tumor size were measured twice every week. At the end of the study mice were sacrificed, and tumors were excised for wet weight, Western blot and H&E staining analyses.

For toxicity study, internal organs including liver and lung were collected at euthanasia for evaluation. Blood was also collected at euthanasia for determination of white blood cell counts and hemoglobin level using vetscan HM5 hematology analyzer (Zoetis Diagnostics, Parsippany, NJ).

### Synergy analysis

Cell-based synergy analysis was performed using the SynergyFinder (Ianevski et al., 2022). Briefly, C4–2, PC-3, DU-145 or DU-145^Doc^ cells were plated at a density of 2000–3000 cells/well in 96-well clear-bottom plates and incubated overnight. They were then treated with docetaxel at 0–62 nM for C4–2, PC-3, and DU14, and 0–6.2 μM for DU-145^Doc^ cells, together with 7I10 at 0–400 nM for C4–2, 0–600 nM for PC-3, 0–350 nM for DU-145, and 0–700 nM for DU-145^Doc^ cells, or 7I14 at 0–400 nM for C4–2, 0–900 nM for PC-3 and DU-145, and 0–1000 nM for DU-145^Doc^ cells. After treatment, cells were subjected to methylene blue assay as described above followed by analysis using the SynergyFinder.

The *in-vivo* tumor growth inhibition synergism was determined using the Bliss independence model (Bliss, 1939). Firstly, the tumor growth inhibition (%) of each treatment group was derived by normalizing tumor wet weight against that in the control treatment group. The expected additive combination inhibition (*I*_*C(E)*_) in % was calculated using the Bliss independence model formula *I*_*C(E)*_=*I*_*7I14(O)*_+*I*_*Doc(O)*_*-I*_*7I14(O)*_×*I*_*Doc(O)*_, where *I*_*7I14(O)*_ and *I*_*Doc(O)*_ represent observed (O) tumor growth inhibition in % by 7I14 and docetaxel as single agent, respectively. The combination is synergistic when the observed combination inhibition of tumor growth (*I*_*C(O)*_) is more than *I*_*C(E)*,_ and additive if *I*_*C(O)*_=*I*_*C(E)*,_ and antagonistic if *I*_*C(O)*_<*I*_*C(E)*._

To determine the synergism of the combination on inducing caspase 3 activation in xenograft tumors, we performed adaptive normalization as described previously (Josephraj et al., 2026) using the Bliss-Compatible Scaling Formula f= (FC-1)/(FC_max_-1), where f represents fractions of maximum caspase 3 activation and FC represents fold change and FC_max_ represents the maximum observed fold change of caspase 3 activation in a treatment group compared with the vehicle control group. The f for each treatment group was then used to calculate the expected combination additive effect using the Bliss independence model as described above with the formula: *f*_*C(E)*_=*f*_*7I14(O)*_+*f*_*Doc(O)*_*-f*_*7I14(O)*_×*f*_*Doc(O)*_, where *f*_*C(E)*_ represents the expected fraction of additive combination induction of caspase 3 activation, *f*_*7I14(O)*_ and *f*_*Doc(O)*_ represent observed (O) fraction of induced caspase 3 activation in % by 7I14 and docetaxel as single agent, respectively. The combination is synergistic if the observed combination induction of caspase 3 (*f*_*C(O)*_) is more than *f*_*C(E)*,_ and additive if *f*_*C(O)*_=*f*_*C(E)*,_ and antagonistic if *f*_*C(O)*_<*f*_*C(E)*._

### Statistical analyses

All statistical analyses were performed based on at least 3 independent experiments. Data were presented as mean ± standard deviation (SD). Student *t*-test was used between two groups; one sample *t*-test was used when treated groups were normalized to control (as 1 or 100); and one-way ANOVA with multiple comparison was used for experiments having more than two unnormalized groups. All p-values were calculated with p ≤ 0.05 considered statistically significant.

## Results

### Identification of the improved leads

**Fig. 1.**
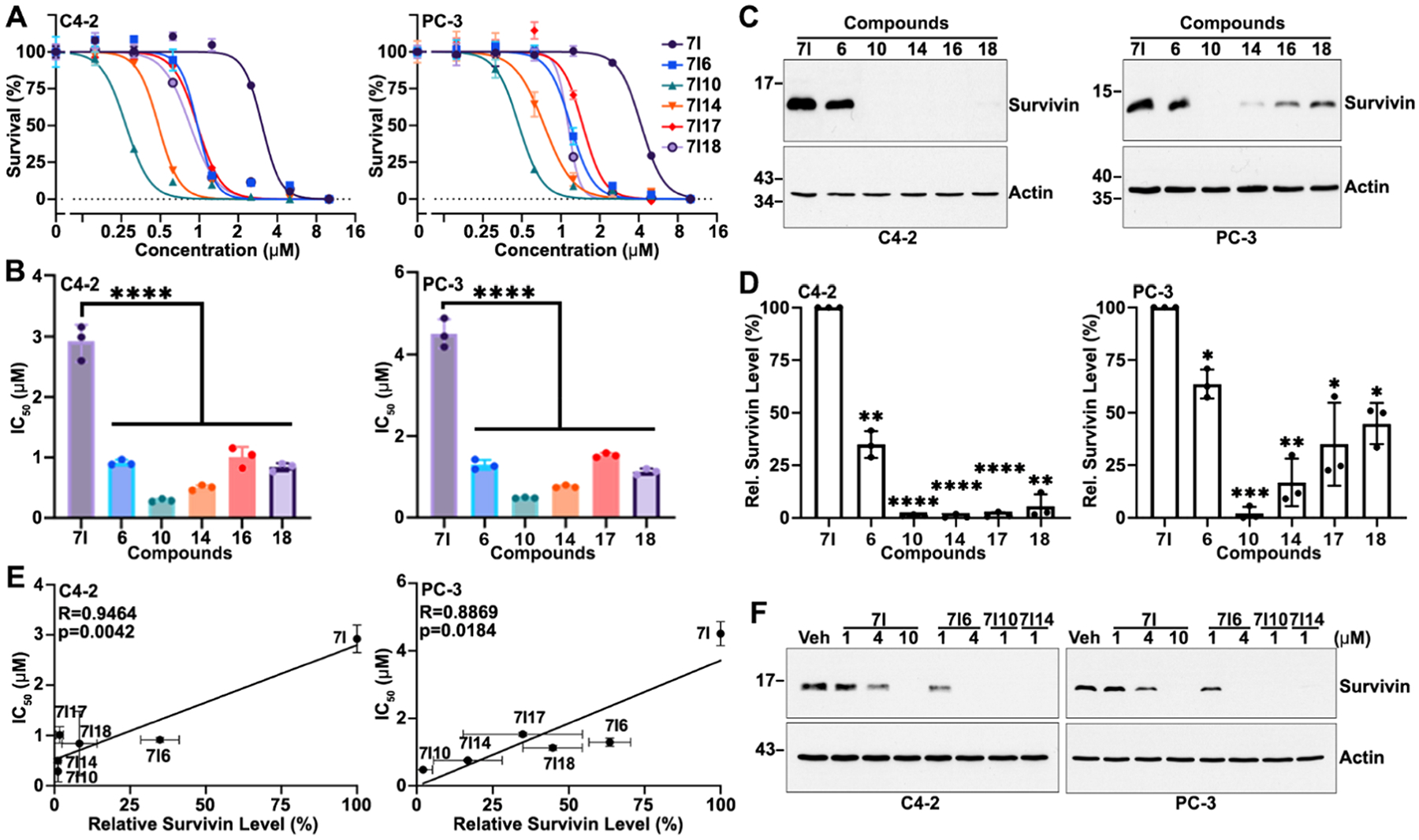
Identification of improved lead compounds. (**A**-**B**) Concentration dependent survival curves of C4–2 and PC-3 cells following 72-hour treatments with different concentrations of 7I and its five analogs (A) and IC_50_ values (B) of these compounds determined from the curves in panel A. The color codes in panel A corresponds to that in panel B. **(C-D)** Western blot analysis of survivin or actin loading control in C4–2 and PC-3 cells following 48-hour treatments with 1 μM 7I or its analogs (C) and quantification of relative survivin protein levels in panel C of three independent experiments (D). **(E)** Pearson correlation analysis between IC_50_ values from panel B and relative survivin protein levels from panel D in C4–2 and PC-3 cells. **(F)** Western blot analysis of survivin and actin loading control in C4–2 and PC-3 cells following treatments with different concentrations of 7I or 7I6 compared with 1 μM 7I10 or 7I14. (n = 3, *p ≤ 0.05, **p ≤ 0.01, ***p ≤ 0.001, and ****p ≤ 0.0001).

The five newly synthesized analogs of LQZ-7I (7I) (Supplemental Figure S1) were first tested to validate their improved potency in inhibiting viability of C4–2 and PC-3 CRPC cells. As shown in Fig. 1A–B and Table 1, all five analogs suppressed both C4–2 and PC-3 cells with significantly improved IC_50_ values ranging from 0.29 ± 0.02–1.29 ± 0.12 μM compared with the parent 7I with IC_50_ values ranging from 2.92 ± 0.27–4.51 ± 0.36 μM, which are consistent with our previous findings (Cui et al., 2025).

To ensure that these new analogs target survivin and induce its loss, we treated both C4–2 and PC-3 cells with each analog at 1 μM and performed Western blot analyses of survivin. As shown in Fig. 1C–D, all five analogs significantly reduced survivin expression in both cell lines compared with their parent 7I, consistent with their increased activity in inhibiting the viability of these cells.

We next conducted a Pearson correlation analysis between their activity in inhibiting cell viability (IC_50_) and the levels of remaining survivin after the treatments. As shown in Fig. 1E, significant (*p* < 0.05) and strong (Pearson’s *r* > 0.7) positive correlations were observed in both cell lines, with the smaller IC_50_ the less survivin remaining. Thus, the potency of these new analogs in inhibiting cell viability is likely related to their potency in inducing survivin loss.

From above analyses, we concluded that 7I10 and 7I14 are most potent while 7I6 the least one among the five active analogs. To validate these conclusions, we treated both C4–2 and PC-3 cells with the parent 7I and the derivative 7I6 at higher concentrations and compared with 7I10 and 7I14 at 1 μM followed by Western blot analyses of survivin level. As shown in Fig. 1F, 10 μM 7I and 4 μM 7I6 are required to achieve the complete elimination of survivin comparable with 1 μM 7I10 and 7I14. These data clearly demonstrate the differential potency of the newly synthesized analogs compared with the parent 7I. Based on the above findings, 7I10 and 7I14 were selected for further evaluations, aiming at delineating their mechanism of action.

### Dose-dependent effects of 7I10 and 7I14 on survivin protein level

**Fig. 2.**
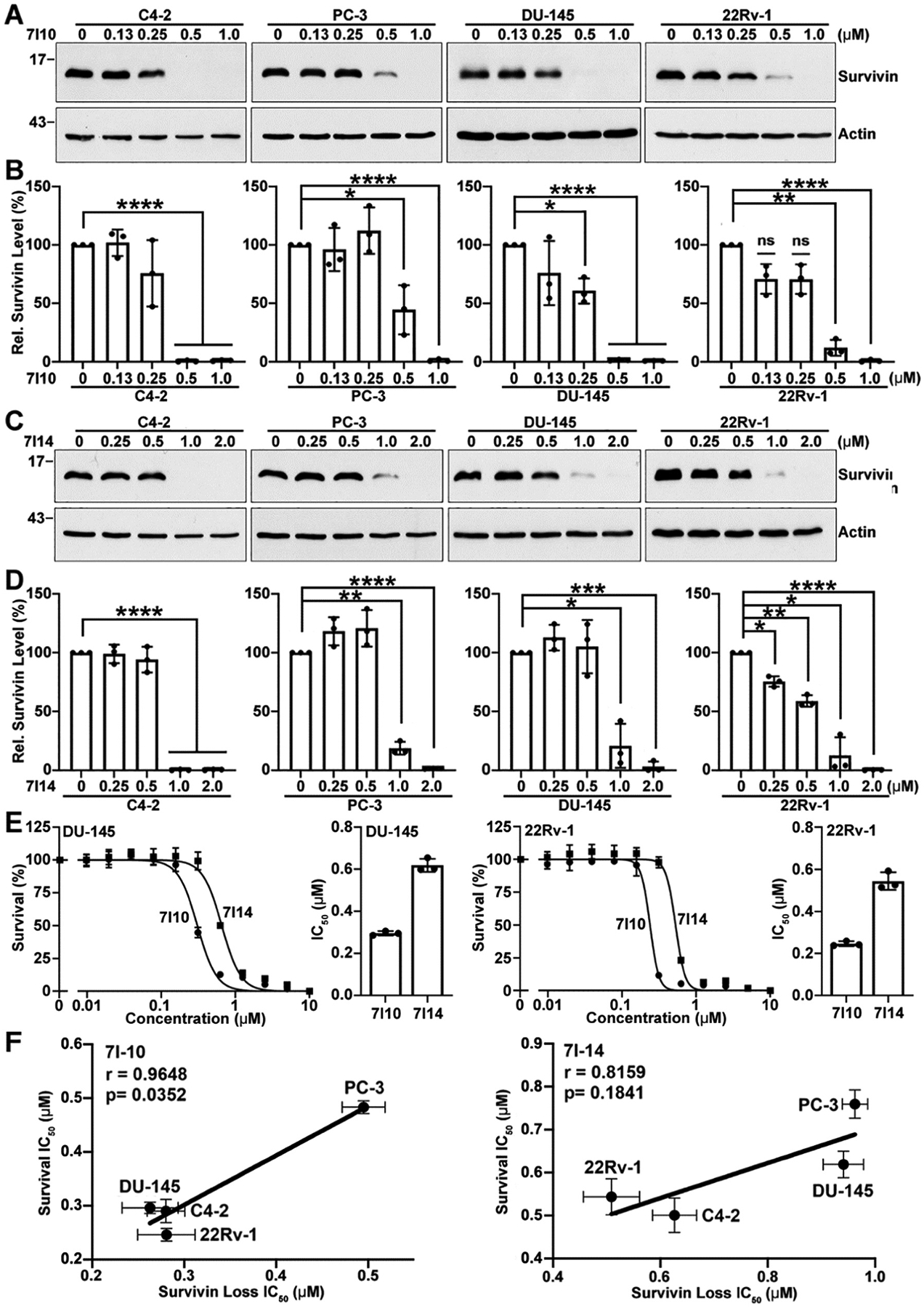
Concentration-dependent effects of 7I10 and 7I14 on survivin expression. (**A** and **C**) Western blot analysis of survivin and actin loading control in C4–2, PC-3, DU-145, 22Rv-1 cells following treatments with different concentrations of 7I10 (A) or 7I14 (C) for 48 h. (**B** and **D**) Quantification of relative survivin protein levels determined from panels A and C. **(E)** Concentration-dependent survival curves of DU-145 and 22Rv-1 cells following 72-hour treatments with different concentrations of 7I10 or 7I14 and IC50 values of 7I0 and 7I14 determined from the curves of three independent experiments. **(F)** Pearson correlation analysis between the survival IC_50_ values of 7I10 or 7I14 from Fig. 1 and Fig. 2E and IC_50_ values of 7I10 or 7I14 causing 50 % loss of survivin calculated from Figs. 2B and 2D. (n = 3, *p ≤ 0.05, **p ≤ 0.01, ***p ≤ 0.001, and ****p ≤ 0.0001).

We next evaluated the dose-dependent effects of 7I10 and 7I14 on survivin expression in C4–2 and PC-3 cells using Western blot analysis. As shown in Fig. 2A–D, the induction of survivin loss is concentration dependent, and 7I10 and 7I14 completely eliminated survivin at 0.5 and 1 μM, respectively. We also extended this test to two additional CRPC cell lines, DU-145 and 22Rv-1. Similarly, both 7I10 and 7I14 induced survivin loss in a concentration-dependent manner and less 7I10 is required than 7I14 to eliminate survivin (Fig. 2A–D). These findings suggest that 7I10 is more potent than 7I14 in eliminating survivin, consistent with their differences in IC_50_ values (see above).

We next measured the cytotoxic IC_50_ of 7I10 and 7I14 against DU-145 and 22Rv-1 cells. Consistent with their IC_50_ values against C4–2 and PC-3 cells, both 7I10 and 7I14 are potent in suppressing the viability of DU-145 and 22Rv-1 cells and 7I10 is significantly more potent than 7I14 (Fig. 2E). With data from four CRPC cell lines, we performed a Pearson correlation analysis between cytotoxic IC_50_ and survivin loss induction IC_50_. As shown in Fig. 2F, strong correlations (Pearson’s *r* > 0.7) were observed, supporting the argument that 7I10 and 7I14 inhibit CRPC cell survival by inducing survivin loss and suggesting that different cells may respond to them differently depending on the endogenous survivin level in these cells (Peery et al., 2020).

### Selectivity of 7I10 and 7I14

**Fig. 3.**
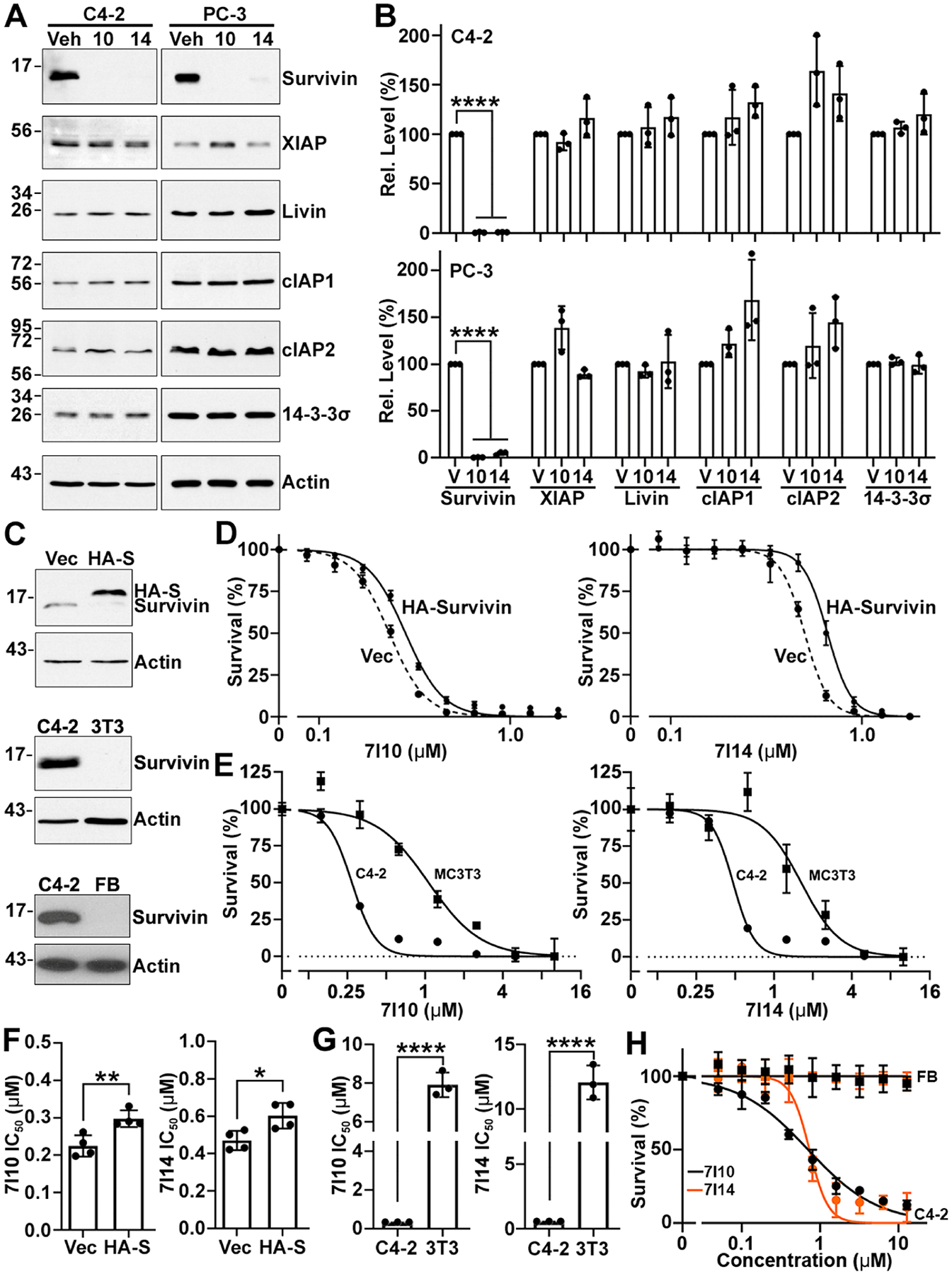
Selectivity of 7I10 and 7I14. (**A**) Western blot analysis of survivin, XIAP, Livin, cIAP1, cIAP2, 14–3–3σ and actin loading control in C4–2 and PC-3 cells following 48-hour treatments with 1 μM 7I10 or 7I14. **(B)** Quantification of relative survivin protein levels in C4–2 and PC-3 cells from panel A of three different experiments. (**C**) Western blot analysis of survivin and actin loading control in stable C4–2 cells overexpressing ectopic HA-tagged survivin (HA-S) or harboring vector control (Vec) (top), MC3T3-E1 cells (3T3) (middle) or primary normal human dermal fibroblasts (FB) (bottom) compared with C4–2 cells. (**D**-**E**) Representative concentration-dependent survival curves of stable C4–2 cells overexpressing HA-tagged survivin (HA-Survivin) or harboring vector control (Vec) (D) and C4–2 and MC3T3-E1 cells (E) following 72-hour treatments with different concentrations of 7I10 or 7I14. (**F**-**G**) IC_50_ values of 7I10 or 7I14 in C4–2 cells harboring vector control or overexpressing HA-survivin (HA-S) (F) and in MC3T3-E1 compared with C4–2 cells (G), determined from survival curves of three different experiments as shown in panels D and E, respectively. (**H**) Representative concentration-dependent survival curves of normal human dermal fibroblast (FB) and C4–2 cells following 72-hour treatments with different concentrations of 7I10 and 7I14. (n = 3, *p ≤ 0.05, **p ≤ 0.01, and ****p ≤ 0.0001).

To investigate the selectivity of 7I10 and 7I14, we first performed Western blot analyses of their effects on the expression of other IAP members. As shown in Fig. 3A–B, among the tested IAPs, survivin was the only protein that was eliminated by 7I10 and 7I14. None of the other IAPs including XIAP, Livin, and c-IAP1/2 was significantly affected. Thus, 7I10 and 7I14 are selective to survivin among the IAP family proteins tested. We also tested the effect of 7I10 and 7I14 on 14–3–3σ, an unrelated but similar homodimeric protein with hydrophobic dimeric interface (Li et al., 2009; Liu et al., 2011). As shown in Fig. 3A–B, 7I10 and 7I14 treatments had no effects on 14–3–3σ expression. Together, these findings indicate that 7I10 and 7I14 are selective to survivin.

To validate above findings, we overexpressed ectopic HA-tagged survivin in C4–2 cells (Fig. 3C) and tested the effect of HA-survivin overexpression on cellular response to 7I10 and 7I14. We expect that overexpressing the survivin target will increase cellular resistance to 7I10 and 7I14. Indeed, the IC_50_ of both 7I10 and 7I14 in C4–2 cells with ectopic survivin expression is significantly increased compared with the control cells (Figs. 3D and 3F).

We also tested a survivin-null cell line, MC3T3-E1, for potential off-target effects of 7I10 and 7I14. Because survivin is essential for survival of cancer cells, there are no survivin-null CRPC cells for such purpose. However, the immortalized mouse osteoblast, MC3T3-E1 cells, show no detectable survivin expression (Fig. 3C), providing an appropriate model to test the potential off-target effects of 7I10 and 7I14. As shown in Figs. 3E and 3G, the IC_50_ of 7I10 and 7I14 is dramatically (~24–27 fold) higher for MC3T3-E1 cells than C4–2 cells, confirming that the effects of 7I10 and 7I14 on cell viability is mainly via acting on survivin.

To further evaluate the selectivity, we tested the impact of 7I10 and 7I14 on the viability of human primary normal fibroblast cells, which also do not express endogenous survivin (Fig. 3C). As shown in Fig. 3H, 7I10 and 7I14 had little effect on the survival of these fibroblasts and we are unable to generate an IC_50_ for either 7I10 or 7I14 in the concentration range tested. These findings confirm that 7I10 and 7I14 have little off-target cytotoxicity against human normal cells, indicating their high selectivity toward survivin.

### 7I10 and 7I14 induction of survivin degradation without effect on its mRNA level

**Fig. 4.**
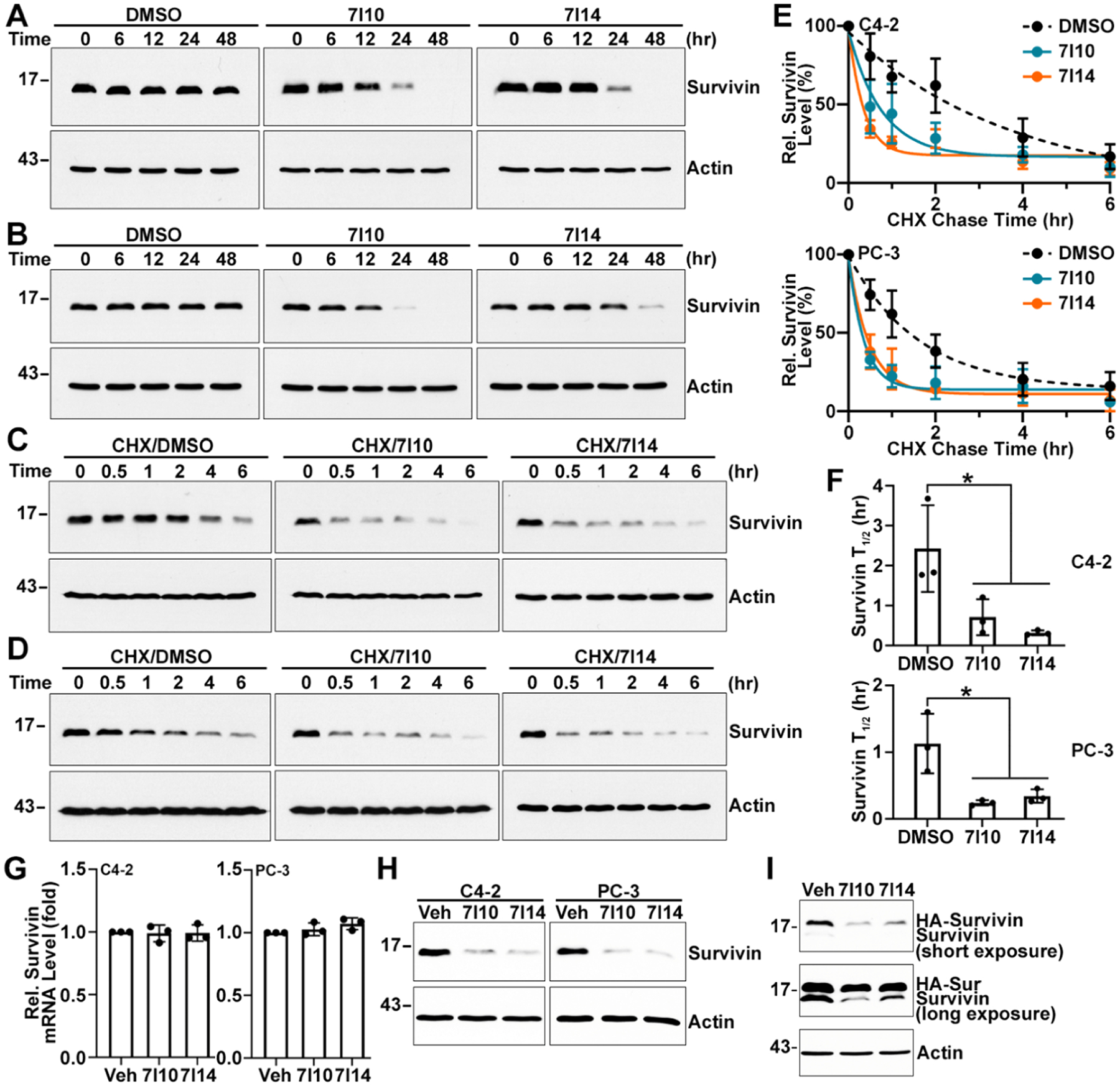
Time-dependent effects of 7I10 and 7I14 on survivin expression and survivin half-life. (**A-B**) Western blot analysis of survivin and actin loading control in C4–2 (A) and PC-3 (B) cells following treatments with 1 μM 7I10 or 7I14 for different times. (**C-D**) Western blot analysis of survivin and actin loading control of C4–2 (C) and PC-3 (D) cells following treatments with 2 μM 7I10 or 7I14 in the presence of 10 μM cycloheximide (CHX) for different times. DMSO vehicle was used as controls for all experiments. **(E-F)** Survivin decay curves (E) and half-life (t_1/2_) (F) as determined by one-phase decay modeling of relative survivin protein level from panels C-D of three independent experiments (*p ≤ 0.05). **(G-H)** Real-time RT-PCR (G) and Western blot (H) analyses of survivin mRNA and protein level in C4–2 and PC-3 cells following 6-hour treatment with 8 μM 7I10 or 7I14. **(I)** Western blot analysis of survivin and actin loading control in stable C4–2 cells overexpressing ectopic HA-survivin driven by CMV promoter following 24-hour treatments with 1 μM 7I10 or 7I14. (n = 3, *p ≤ 0.05).

To understand more about the mechanism of 7I10 and 7I14-induced survivin protein loss, we first analyzed the time-course of survivin loss. As shown in Figs. 4A–B, 1 μM 7I10 and 7I14 were able to eliminate survivin in 48 h in both C4–2 (Fig. 4A) and PC-3 (Fig. 4B) cells, with 50 % reduction in less than 24 h.

Next, we tested the possibility that 7I10 and 7I14 induce survivin loss by destabilizing survivin due to inhibition of its dimerization by performing cycloheximide chase assay to determine survivin half-life (t_1/2_) in the absence or presence of 7I10 or 7I14. As shown in Fig. 4C–F, survivin has a normal t_1/2_ of 2.43 ± 0.89 h and 1.13 ± 0.37 h in C4–2 and PC-3 cells, respectively, consistent with previous findings (Peery et al., 2020; Qi et al., 2016). In the presence of 2 μM 7I10 or 4 μM 7I14, the survivin t_1/2_ was significantly shortened to 0.71 ± 0.37 and 0.32 ± 0.05 h in C4–2 cells, and 0.24 ± 0.03 and 0.34 ± 0.08 h in PC-3 cells, respectively.

To eliminate the possibility that 7I10 and 7I14-induced survivin loss was due to inhibition of survivin gene transcription, we performed real-time RT-PCR analysis following 7I10 and 7I14 treatments. To eliminate potential secondary effects, we challenged C4–2 and PC-3 cells with 8 μM 7I10 or 7I14 for 6 h followed by Western blot and real-time RTPCR analyses. As shown in Fig. 4G–H, the survivin mRNA level was not affected while its protein was dramatically reduced under the same condition. Thus, we conclude that 7I10 and 7I14 are likely to work directly on survivin protein, not its gene transcription. Additionally, 7I10 and 7I14 reduced the expression of ectopic HA-survivin in C4–2 cells (Fig. 4I), which is driven by CMV promoter and not by native survivin promoter, further confirming the above conclusion.

### 7I10 and 7I14 induction of proteasome-dependent survivin degradation

**Fig. 5.**
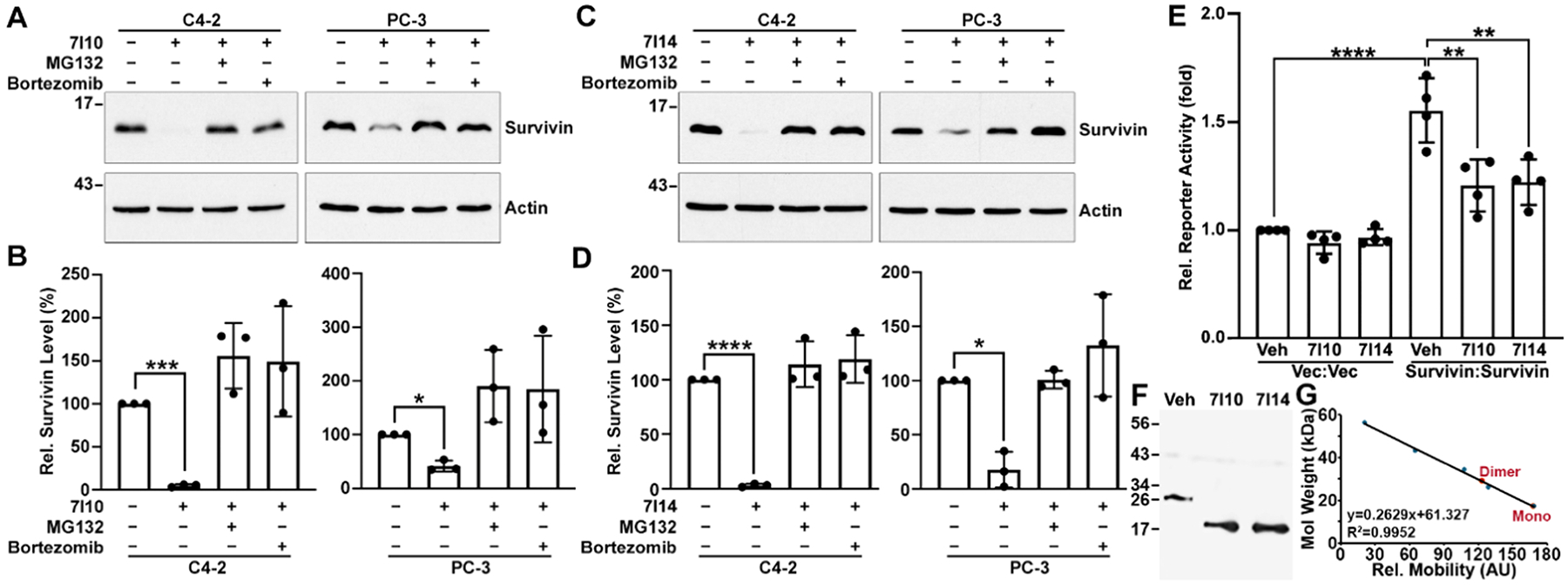
Proteasome-dependent survivin degradation and inhibition of dimerization. (**A** and **C**) Western blot analysis of survivin and actin loading control in C4–2 and PC-3 cells following treatments with 1 μM of 7I10 (A) or 7I14 (C) in the absence or presence of proteasome inhibitors MG132 (10 μM) or bortezomib (70 nM). (**B** and **D**) Quantification of relative survivin protein levels from panels A and C of three independent experiments. **(E)** SEAP reporter activity driven by survivin dimerization as determined using mammalian two hybrid assay in the presence of vehicle or 2 μM 7I10 or 7I14. (n = 3, *p ≤ 0.05, **p ≤ 0.01, ***p ≤ 0.001, and ****p ≤ 0.0001). (**F**) PFO-PAGE and Western blot analysis of survivin. C4–2 cells were treated with 0.6 ng/mL docetaxel followed by treatment with vehicle, 2 μM 7I10, or 4 μM 7I14 in the presence of 10 μM MG132 followed by lysis using PFO lysis buffer and analyzed using PFO-PAGE and Western blot probed with survivin antibody. (**G**) The mobility of survivin was analyzed against that of molecular weight markers to derive the apparent molecular weight of survivin from panel F. AU, arbitrary unit.

Next, we tested the possibility that 7I10 and 7I14 caused survivin degradation in proteasome by performing a rescue experiment using proteasome inhibitors MG132 and bortezomib. As shown in Fig. 5A–D, pre-treatment with MG132 or bortezomib completely reversed 7I10 and 7I14-induced survivin degradation in both C4–2 and PC-3 cells.

### 7I10 and 7I14 inhibition of survivin dimerization

To determine that survivin degradation in proteasome was due to 7I10 and 7I14 disruption of survivin homodimerization, we performed a mammalian two-hybrid assay in the absence or presence of 7I10 and 7I14 as previously reported (Peery et al., 2020). In this assay, survivin was fused with the GAL4-DNA binding domain in one construct and the VP16 transactivation domain in another. The interaction between these two domains as a result of survivin dimerization activates the expression of the secreted alkaline phosphatase (SEAP) reporter gene. As shown in Fig. 5E, survivin dimerization (Survivin:Survivin) in the presence of vehicle activated the expression of SEAP compared with vector (Vec: Vec) control as expected. However, survivin dimerization-induced SEAP expression was significantly inhibited in the presence of 7I10 or 7I14, indicating that survivin dimerization was disrupted by 7I10 and 7I14.

To validate above findings, we adapted PFO-PAGE to analyze the dimerization status of survivin following 7I10 and 7I14 treatments. PFO, a non-denaturing ionic detergent, has previously been used to extract and preserve oligomeric membrane proteins for size analysis using PFO-PAGE (Xu et al., 2004). As shown in Fig. 5F–G, the apparent molecular weight of survivin in cells treated with 2 μM 7I10 or 4 μM 7I14 is 16.9 kDa, equivalent to the size of a monomeric survivin, as determined using linear regression of molecular weight standards. It has an estimated molecular weight of 28.5 kDa in cells treated with vehicle control. While this is slightly less than the size of a dimer, the molecular weight of monomeric survivin may be over-estimated due to 7I10/7I14 binding, which not only increases the molecular weight, but also decreases mobility of monomeric survivin due to added positive charges. Thus, these results confirm that 7I10 and 7I14 are potent inhibitors that disrupt survivin dimerization.

### 7I10 and 7I14 induction of spontaneous apoptosis

**Fig. 6.**
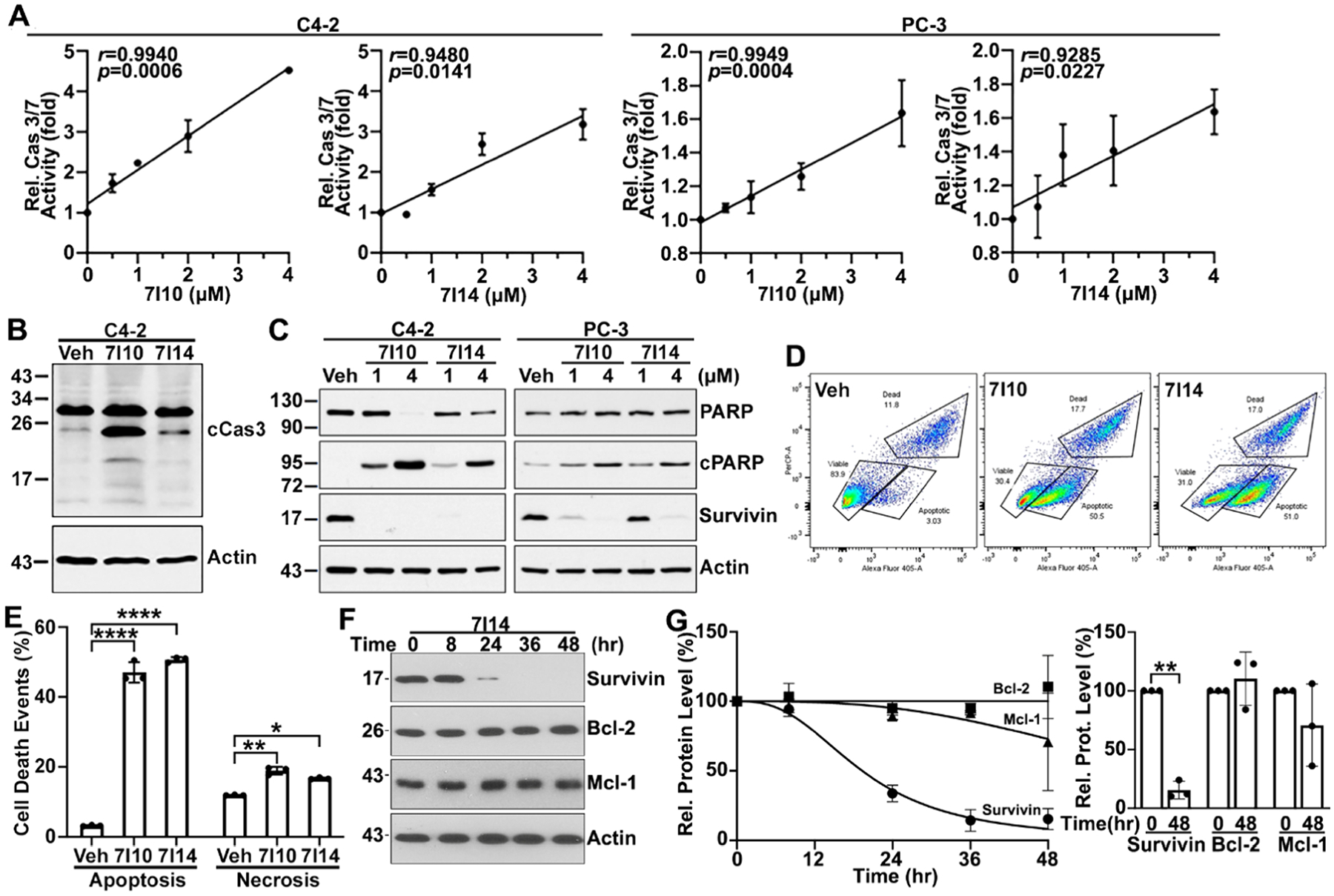
7I10- and 7I14-induced spontaneous apoptosis. (**A**) Caspase 3/7 activity in C4–2 and PC-3 cells following 24-hour treatments with 7I10 or 7I14 at different concentrations as determined using the Caspase-Glo 3/7 activity assay (Promega). **(B)** Western blot analysis of cleaved caspase 3 and actin loading control in C4–2 cells following 48-hour treatment with 1 μM 7I10 or 7I14. **(C)** Western blot analysis of cleaved PARP1, survivin, and actin loading control in C4–2 and PC-3 cells following 24-hour treatments with 1 or 4 μM 7I10 or 7I14. **(D)** Flow cytometry-based Vybrant^®^ DyeCycle^™^ analysis of PC-3 cells following 24-h treatment with 4 μM 7I10 or 7I14 with 20,000 events acquired for each condition and gated for three distinct populations. **(E)** Cell death event (%) analysis of the gated populations from panel D of three independent experiments. (**F**) Western blot analysis of Bcl-2 and Mcl-1 compared with survivin with actin loading control following 7I14 treatments for different times in C4–2 cells. (**G**) Quantification of relative survivin, Bcl-2, and Mcl-1 proteins from panel F of three independent experiments and comparison between 0 and 48 h data points. (n = 3, *p ≤ 0.05, **p ≤ 0.01, and ****p ≤ 0.0001).

Inhibition of survivin is known to induce spontaneous apoptosis (Tamm et al., 1998). Thus, we next tested the effect of 7I10 and 7I14 on apoptosis. For this purpose, we first determined caspase-3/7 activation following 7I10 and 7I14 treatments in C4–2 and PC-3 cells by measuring caspase-3/7 activities using the Promega Caspase-Glo^®^ 3/7 Assay kit. As shown in Fig. 6 A, the caspase-3/7 activities were dose-dependently increased by 7I10 and 7I14 treatments, indicating that both 7I10 and 7I14 induce spontaneous apoptosis in these cells. Western blot analysis of cleaved caspase 3 in C4–2 cells confirms caspase 3 activation by both 7I10 and 7I14 (Fig. 6B).

We next determined PARP cleavage as another indicator of apoptosis (Boulares et al., 1999; Mashimo et al., 2021) following 7I10 and 7I14 treatments. As shown in Fig. 6 C, both 7I10 and 7I14 dose-dependently induced PARP cleavage in both C4–2 and PC-3 cells as determined using Western blot analysis, consistent with the observation of caspase-3/7 activation by 7I10 and 7I14 (Fig. 6 A). Indeed, both 7I10 and 7I14 induced about 50 % spontaneous apoptosis of PC-3 cells as determined using a flow cytometry-based assay of condensed heterochromatins (Fig. 6D–E). Together, these results show that 7I10 and 7I14 induce spontaneous apoptosis, consistent with their activity in inducing survivin degradation.

To further understand the mechanism of apoptosis induction, we examined the effects of 7I14 as a representative compound on the expression of other anti-apoptotic proteins Bcl-2 and Mcl-1 in addition to other IAP members (see Fig. 3). As shown in Fig. 6F–G, the expression of both Bcl-2 and Mcl-1 is unaffected by 7I14 up to 48-hours treatment, whereas that of survivin was eliminated. Thus, the induction of apoptosis by our survivin degraders such as 7I14 is likely directly through survivin and these findings further support the conclusion that 7I14 is selective in targeting survivin.

### Both 7I10 and 7I14 synergize with docetaxel

**Fig. 7.**
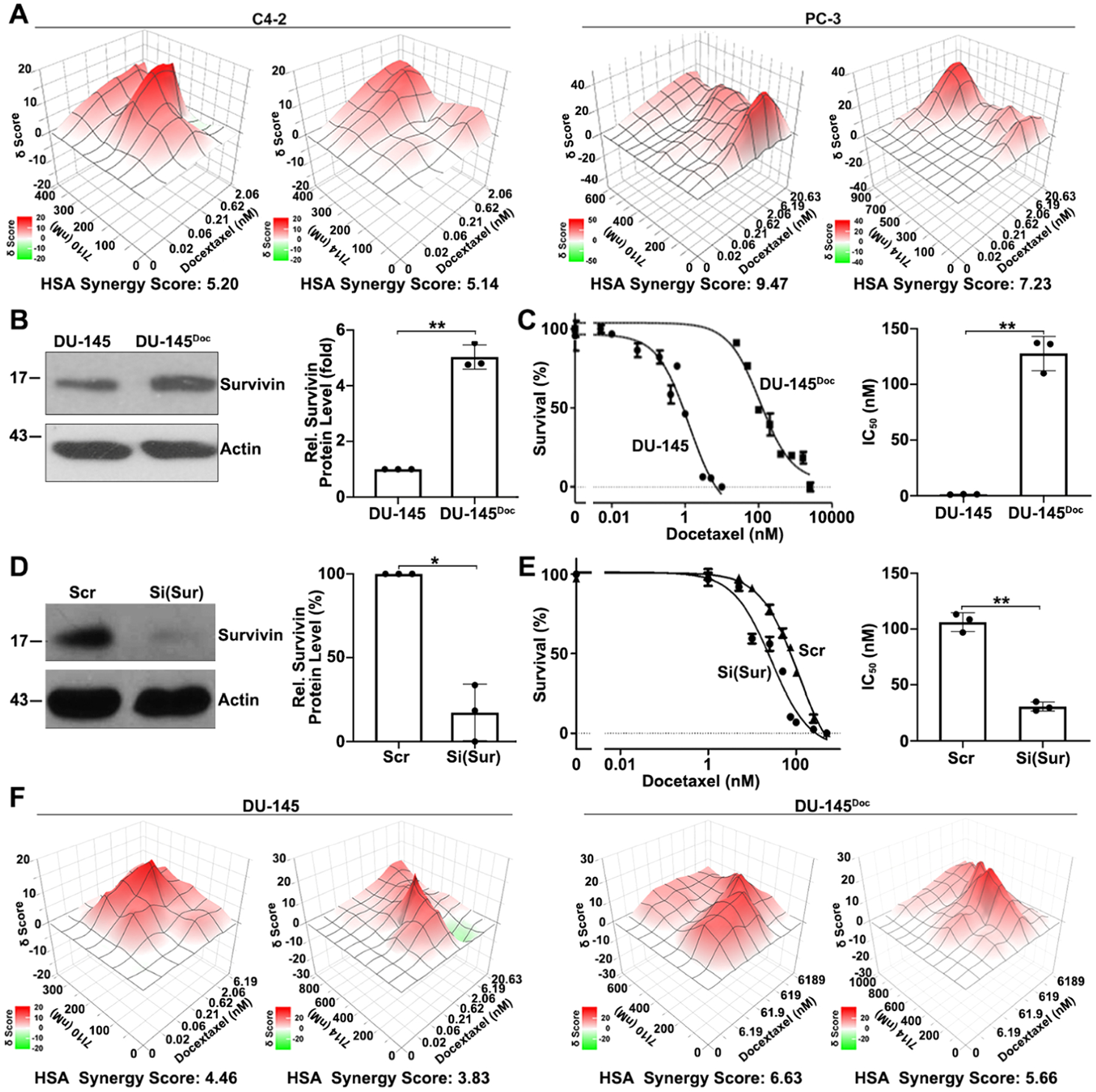
7I10 and 7I14 synergize with docetaxel and overcome docetaxel resistance. **(A and F)** The synergistic effect between docetaxel and 7I10 or 7I14 on survival of C4–2 and PC-3 cells (A) or DU-145 and DU-145^Doc^ cells (F) as determined using methylene blue survival assay and the data were processed using the SynergyFinder+ program. Positive δ scores (z-axis) indicate synergism. (**B** and **D**) Western blot analysis of survivin and actin loading control in DU-145 and DU-145^Doc^ cells (B) or DU-145^Doc^ cells transiently transfected with scrambled control (Scr) or survivin [Si(Sur)] siRNAs (D). The bar graphs show survivin protein levels in these cells quantified from Western blot of three different experiments. (**C** and **E**) Representative concentration-dependent survival curves of DU-145 and DU-145^Doc^ (C) and DU-145^Doc^ cells harboring scrambled control (Scr) or survivin [Si(Sur)] siRNAs (E). The bar graphs show docetaxel IC_50_ in DU-145, DU-145^Doc^, and DU-145^Doc^ cells harboring scrambled control (Scr) or survivin [Si(Sur)] siRNAs derived from the survival curves.

Docetaxel is one of the first line treatment for metastatic CRPC and survivin is known to contribute resistance to drugs including docetaxel (Ghanbari et al., 2014; Peery et al., 2022; Vinod et al., 2015). To investigate if 7I10 and 7I14 may synergize with docetaxel and help overcome docetaxel resistance by inducing survivin degradation, we performed a combination survival study and analyzed the synergy using the maximum single drug response (HSA) model in a web-based portal SynergyFinder+ (Zheng et al., 2022). As shown in Fig. 7 A, strong synergism was observed between docetaxel and 7I10 or 7I14 in both C4–2 and PC-3 cells, suggesting that both 7I10 and 7I14 may be used to overcome docetaxel resistance in combinational therapy.

To test this possibility, we created docetaxel-resistant DU-145 (DU-145^Doc^) cells by stepwise selection as previously described (Qin et al., 2014). As shown in Fig. 7B–C, DU-145^Doc^ cells express about 5-fold more survivin with a docetaxel IC_50_ of 127.8 nM compared to its parental DU-145 cells with an IC_50_ of 1.3 nM. Suppressing the upregulation of survivin expression in DU-145^Doc^ cells using siRNA (Fig. 7D), significantly dropped docetaxel IC_50_ more than 70 % to ~30 nM (Fig. 7E). These findings suggest that survivin is likely responsible for most of the acquired docetaxel resistance in DU-145^Doc^ cells.

To determine if 7I10 and 7I14 can overcome the acquired docetaxel resistance, we performed combination study as described above using both DU-145 and DU-145^Doc^ cells. As shown in Fig. 7 F, the combination of docetaxel with 7I10 or 7I14 resulted in strong synergism for both cell lines. However, DU-145^Doc^ cells have higher synergy scores for both 7I10 and 7I14 compared with the parental DU-145 cells, suggesting a better synergy for the docetaxel-resistant DU-145 cells. This observation is consistent with the finding on synergy for PC-3 and C4–2 cells. PC-3 cells, which express higher level of endogenous survivin and are more resistant to docetaxel than C4–2 cells (Peery et al., 2020), have higher synergy scores than C4–2 cells (Fig. 7 A). Thus, we conclude that both 7I10 and 7I14 can overcome both intrinsic and acquired docetaxel resistance by inducing survivin degradation.

Because testosterone and androgen receptor (AR) signaling has been implicated in docetaxel resistance in CRPC (Mout et al., 2020) and AR signaling has also been shown to regulate survivin expression, leading to resistance to apoptosis (Zhang et al., 2005), we analyzed the effect of 7I10 and 7I14 on AR signaling by determining the expression of its downstream target gene PSA in C4–2 cells. As shown in supplemental Figure S2, neither 7I10 nor 7I14 affected the expression of AR or PSA. Thus, it is unlikely that AR signaling plays any role in the synergy observed above.

### In-vivo activity and synergism with docetaxel

**Fig. 8.**
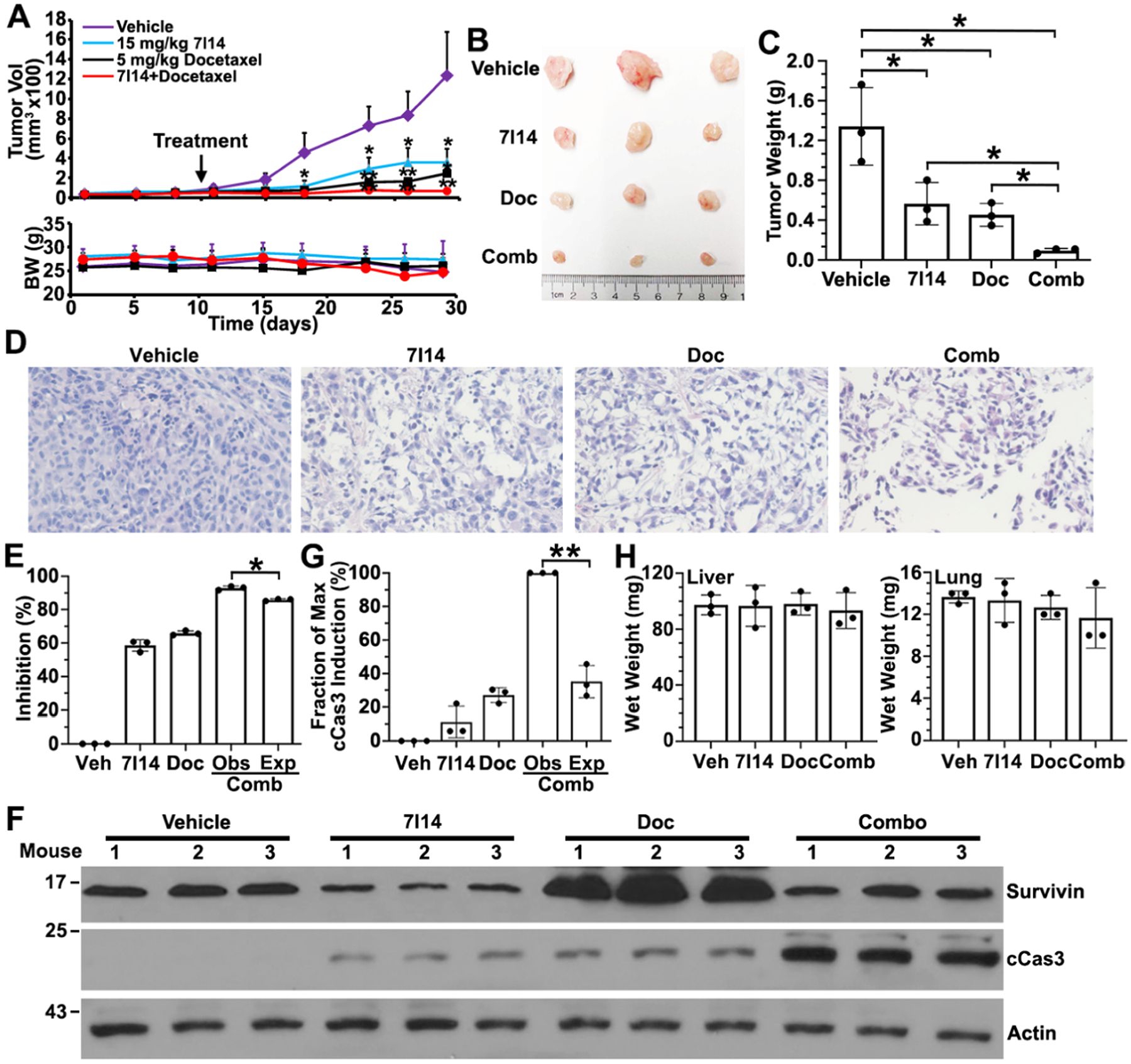
*In-vivo* efficacy and synergism. (**A**) Effect of 7I14, docetaxel (Doc), or combination of the two agents (Comb) on the growth of PC-3 xenograft tumors and body weight (BW) of male NSG mice. **(B)** Gross anatomy of dissected xenograft tumors. **(C)** Final wet weight of xenograft tumors. **(D)** Representative images of H&E-stained xenograft tumor sections. **(E)** Tumor growth inhibition (%) by single agent or combination treatment. The expected (Exp) combination additivity was calculated from the observed inhibition by each single agent alone using the Bliss independence model (see Materials and Methods). Obs, observed combination inhibition. **(F)** Western blot analysis of survivin, cleaved caspase 3 (cCas3), and actin loading control in individual xenograft tumors. **(G)** Synergy analysis of cleaved caspase 3 (cCas3) induction. The cCas3 production was quantified from panel F. The expected combination induction was calculated using the adaptive Bliss independence model (see Materials and Methods). **(H)** Wet weight of liver and lung of the mice in different groups. (n = 3, *p ≤ 0.05, **p ≤ 0.01, and ***p ≤ 0.001).

Finally, we tested the *in-vivo* activity and synergism with docetaxel using 7I14 as the representative lead in combination with docetaxel due to its better solubility than 7I10. For this purpose, PC-3 xenograft tumors were established in NSG mice, followed by treatments with 15 mg/kg 7I14 twice a week, 5 mg/kg docetaxel once a week, or both in combination for three weeks. As shown in Fig. 8A, 7I14 as a single agent significantly slowed the growth of the xenograft tumor similarly as docetaxel alone while the combination completely stopped the tumor growth. The dissected tumors at the end of the treatments are significantly smaller in size (Fig. 8B), wet weight (Fig. 8C), and cancer cell density (Fig. 8D) in the tumors of the combination treatment group, confirming the above conclusion. Further analysis of the inhibitory effect of the combination using the Bliss independence model (Bliss, 1939) revealed a significant synergism between docetaxel and 7I14 in the combination in inhibiting tumor growth (Fig. 8E), consistent with our cell-based observations (Fig. 7).

Analysis of cleaved caspase 3 in the isolated tumors using Western blot indicates that 7I14, similar to docetaxel, induced apoptosis (Fig. 8F). However, the combination clearly induced much more apoptosis. Quantification and analysis of induced cleavage of caspase 3 using the adaptive Bliss independence model (Josephraj et al., 2026) showed that induction of apoptosis by the combination is synergistic (Fig. 8G), consistent with the synergism in inhibiting tumor growth (Fig. 8E).

To determine the *in-vivo* target inhibition, we performed Western blot analyses of survivin in all individual xenograft tumors. As shown in Fig. 8F, 7I14 successfully reduced survivin expression as a single agent or in combination with docetaxel, indicating the *in-vivo* effect of 7I14 on xenograft tumors is likely due to 7I14-induced survivin degradation in the tumors.

Finally, we analyzed the potential toxicity of 7I14. As shown in Fig. 8A, the body weight of mice did not change significantly in the treatment groups compared with the control group, suggesting that no significant toxicity was observed in the treatment groups including the combination group. This conclusion is corroborated by the finding that the wet weight of livers and lungs across all groups did not change significantly (Fig. 8H).

To validate above findings and to ensure scientific rigor, we performed another independent *in-vivo* experiment using 7I14. As shown in Supplemental Figure S3, same findings were observed with the efficacy as a single agent or in combination (Figure S3A–D), the synergism with docetaxel in inhibiting tumor growth (Figure S3E), *in-vivo* induction of survivin loss and cleavage of caspase 3 (Figure S3F), and lack of toxicity as indicated by lack of body weight loss (Figure S3A) and without change in wet weight of liver and lung (Figure S3G). Furthermore, the white blood cell counts, and hemoglobin level remained similar between the treatment and control groups (Figure S3H). Thus, we conclude that 7I14 at 15 mg/kg twice a week and its combination with docetaxel does not cause significant toxicity to mice while synergizing with docetaxel in inhibiting xenograft tumor growth.

## Discussion

In the current study, we characterized in detail the activities and mechanism of action of two novel survivin degraders, 7I10 and 7I14. We demonstrated that both 7I10 and 7I14 are much more potent than their parent 7I in inducing cytotoxicity and proteasome-dependent survivin degradation by inhibiting its dimerization. 7I10 and 7I14 are also selective to survivin and capable of inducing spontaneous apoptosis. Furthermore, both compounds synergize with docetaxel, the first-line treatment for metastatic CRPC, and overcome docetaxel resistance. Moreover, 7I14 is effective in suppressing xenograft tumor growth and synergize with docetaxel *in vivo* without any apparent toxicity.

As discussed above, survivin is a difficult-to-drug target due to lack of enzymatic activities. Many past efforts have been on targeting survivin expression using antisense oligonucleotides or inhibiting its upstream regulators (Cui et al., 2023; Peery et al., 2017). The most notable outcome in these efforts was the discovery of YM155 targeting survivin gene transcription (Nakahara et al., 2007), which led to multiple unsuccessful clinical trials (Cui et al., 2023). This failure was thought to be due to its poor pharmacokinetics and multiple targets including SP1, which is responsible for expression regulation of survivin and many other genes (Cheng et al., 2012). Other small-molecule inhibitors including EM-1421 and FL118 similarly target upstream transcription factors and may suffer from similar drawbacks (Cui et al., 2023). Because their direct targets are transcription factors, not survivin protein itself, naming them survivin inhibitors is misleading (Cui et al., 2023).

There are also efforts in targeting survivin protein with small molecule inhibitors including Abbott 8 and its derivatives (Chettiar et al., 2013; Wendt et al., 2007). However, no recent progress has been reported and none of them are being tested clinically. Targeting survivin interaction with its partners has also been attempted with discovery of UC-112 and its derivatives (Wang et al., 2018). Interestingly, these inhibitors also induced survivin loss although the mechanism is unclear.

Our approach to selectively induce survivin degradation by binding to survivin protein and inhibiting its homodimerization overcomes the issues and drawbacks of the above approaches and the long-standing undruggable challenge in targeting survivin. We are hopeful that 7I10 and 7I14 representing the third-generation survivin degraders will lead us closer to a true survivin inhibitor for further development.

Protein degraders such as PROTAC (proteolysis targeting chimera) have recently been recognized as an effective approach to target undruggable proteins with many in clinical testing (Bekes et al., 2022). However, PROTAC requires linking the ligand targeting the protein of interest to another ligand targeting E3 ligase in order to induce proteasome-dependent degradation of the protein of interest, while 7I10 and 7I14 induce survivin degradation without E3 ligase ligand. Although we have shown that 7I10 and 7I14 induce survivin degradation via proteasome, it is unclear if the binding of 7I10 or 7I14 to survivin induces recruitment of E3 ligase and ubiquitination of survivin for its degradation. It is, however, noteworthy that survivin has previously been shown to be ubiquitinated for degradation and the ubiquitination likely occurs on multiple lysine residues including the ones at the amino terminal domain (Zhao et al., 2000). Deletion of residues 71–142 reduced survivin ubiquitination. The dimerization domain of survivin is also located in the amino terminus including residues 89–102 (Peery et al., 2017), which contains two lysine residues that may get exposed for ubiquitination due to inhibition of dimerization. Clearly, further studies on this possibility are warranted, and we are currently working toward this effort.

It is also noteworthy that the evidence is lacking for the direct binding of 7I10 and 7I14 to survivin although their effects on survivin dimerization and degradation imply their direct interactions. Nevertheless, we have shown previously that LQZ-7, the parent precursor of 7I10 and 7I14, directly binds to survivin (Qi et al., 2016). Thus, we believe that 7I10 and 7I14 likely bind to survivin directly. Future studies are necessary to determine the affinity and kinetics of 7I10 and 7I14 binding to survivin using biophysical methods such as isothermal titration calorimetry.

Although 7I10 and 7I14 are likely selective to survivin protein as evidenced in this study, we cannot exclude the possibility that they may have undetected off-target effects. Additional studies are clearly needed to exclude this possibility. However, examination of 7I10 and 7I14 structures shows that they are unlikely pan-assay interference compounds (PAINS). Furthermore, 7I10 and 7I14 are the third-generation compounds that can inhibit survivin dimerization and induce its degradation. Additionally, other analogs of the parent compound 7I with similar structures to that of 7I10 and 7I14 do not have cytotoxicity or induce survivin loss (Peery et al., 2020). These observations confirm that 7I10 and 7I14 are unlikely PAINS.

Survivin is expressed in essentially all cancer cell lines and immortalized non-cancer cell lines, different from normal tissues. It is, thus, difficult to test the effect of survivin inhibitors on normal or non-cancer human cells. However, immortalized mouse osteoblast cell line, MC3T3-E1, and normal primary human fibroblasts that lack detectable survivin could be used as surrogates to help delineate survivin selectivity and potential off-target effects as shown in this study. Furthermore, lack of toxicity as shown using xenograft animal models indicate that 7I14 at the dose used is safe for further development. Indeed, previously it has been shown that inhibiting survivin expression using inhibitors such as YM-155 by inhibiting SP1 is safe in clinical studies (Cui et al., 2023), consistent with the fact that survivin is expressed in most cancers but not in adult normal tissues.

Because survivin is overexpressed and may be required for survival of all cancer cells and its lack of expression in most adult normal tissues, 7I10 and 7I14 may be developed as therapeutics for many cancers, not just metastatic CRPC as we studied here. Additionally, because survivin as a survival factor has been shown to cause resistance to various cancer therapeutics (Peery et al., 2017) and likely contribute to acquired docetaxel resistance in CRPC cells (Fig. 7), 7I10 and 7I14 may be developed as a combinational therapy to alleviate the resistance to other drugs. Indeed, both 7I10 and 7I14 synergize with docetaxel and overcome acquired docetaxel resistance in cell-based studies, and 7I14 synergizes with docetaxel in a xenograft animal model, which is consistent with a previous study that showed sensitization of breast cancer cells to docetaxel by inhibiting survivin expression (Ghanbari et al., 2014).

Furthermore, it is well established that cancer stemness plays an essential role in drug resistance (Loh and Ma, 2024). Interestingly, survivin has been shown to overexpress in different stem cells including cancer stem cells (Canato et al., 2025; Warrier et al., 2020). Epithelial-mesenchymal transition (EMT) has also been suggested to contribute to drug resistance and inhibiting survivin reverse EMT (Zhang et al., 2018; Zhao et al., 2019). Therefore, the survivin degraders 7I10 and 7I14 may have broader therapeutic potential in overcoming multidrug resistance mediated by cancer stem cells and EMT and, thus, warrant further investigation.

In summary, two new quinoxaline-based survivin dimerization inhibitors, 7I10 and 7I14 have been identified that directly target survivin protein and inhibit survivin dimerization, leading to its degradation in proteasome and spontaneous apoptosis. These inhibitors also synergize with docetaxel and are effective in suppressing xenograft tumor growth without apparent toxicity at the dose used. We believe that these new survivin degraders promise a new direction of drug discovery targeting “undruggable” homodimeric proteins and development of novel cancer therapeutics as single agents and for combinational therapy.

## Supplementary Material

1

## Figures and Tables

**Table 1:** IC50 of LQZ-7 and its derivatives in C4–2 and PC-3 cells.

Compounds	IC_50_ (μM)	
	C4-2	PC-3
LQZ-7I	2.92 ± 0.27	4.51 ± 0.36
LQZ-7I6	0.92 ± 0.05	1.29 ± 0.12
LQZ-7I10	0.29 ± 0.02	0.48 ± 0.01
LQZ-7I14	0.50 ± 0.04	0.76 ± 0.03
LQZ-7I17	1.01 ± 0.09	1.53 ± 0.05
LQZ-7I18	0.84 ± 0.06	1.13 ± 0.07

## Data Availability

The data generated in this study are available within the article and its supplementary data files.

## Appendix A. Supporting information

Supplementary data associated with this article can be found in the online version at doi:10.1016/j.drup.2026.101356.
