# Supplementary material for: Novel quinoxaline-based survivin degraders overcome docetaxel-resistance in castration-resistant prostate cancer
Source: Drug Resist Updat. Author manuscript; Available in PMC 2026 Jun 29. (PMC13312427; doi:10.1016/j.drup.2026.101356)
Supplement: 1 [file NIHMS2189680-supplement-1.pdf]

Novel quinoxaline-based survivin degraders overcome docetaxel resistance in castration-resistant prostate cancer

Caoqinglong Huang, Qingbin Cui, Xunzhen Zheng, Robert C. Peery, Zizheng Dong, Xiaohong Li, Jing-Yuan Liu*, Jian-Ting Zhang*

Department of Cell and Cancer Biology, University of Toledo College of Medicine and Life Sciences, Toledo, OH 43614, USA

*Corresponding authors: Email: jingyuan.liu@utoledo.edu and jianting.zhang@utoledo.edu.

Supplemental Figures

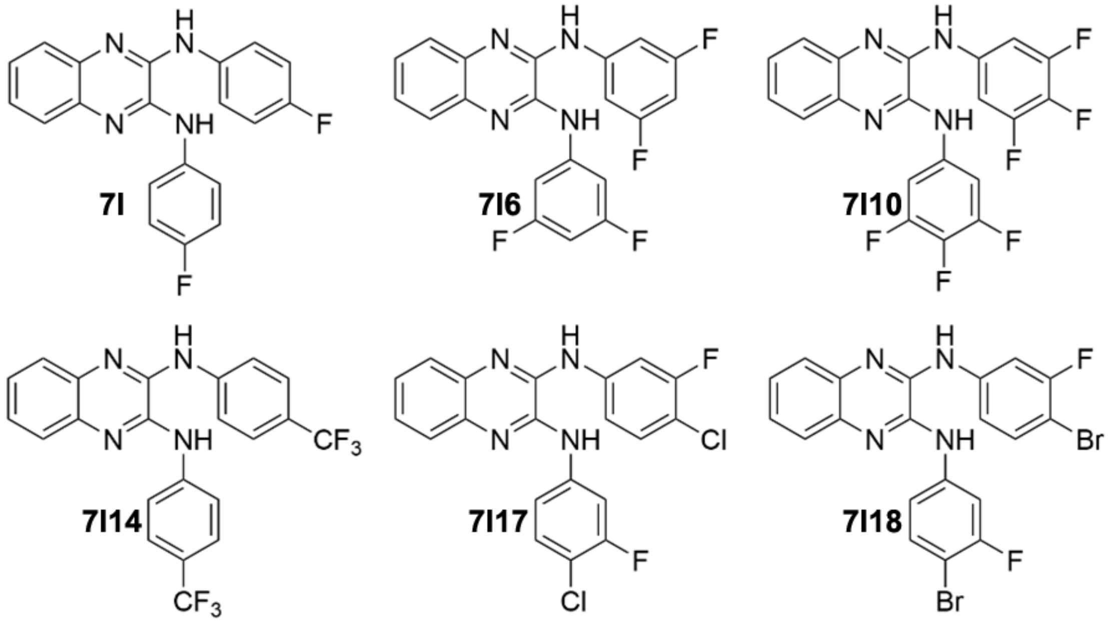

Figure S1. Chemical structures of LQZ-7I (7I) and its active derivatives.

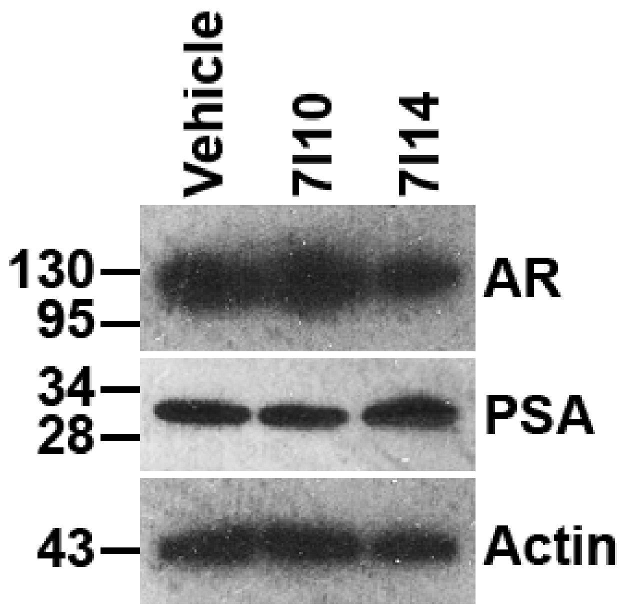

Figure S2. Effect of 7I10 and 7I14 on androgen receptor and prostate-specific antigen expression. C4-2 cells were treated with DMSO vehicle, 1 μM 7I10, or 2 μM 7I14 for 24 hours before total lysate preparation and western blot analysis of androgen receptor (AR), prostate-specific antigen (PSA), and actin loading control.

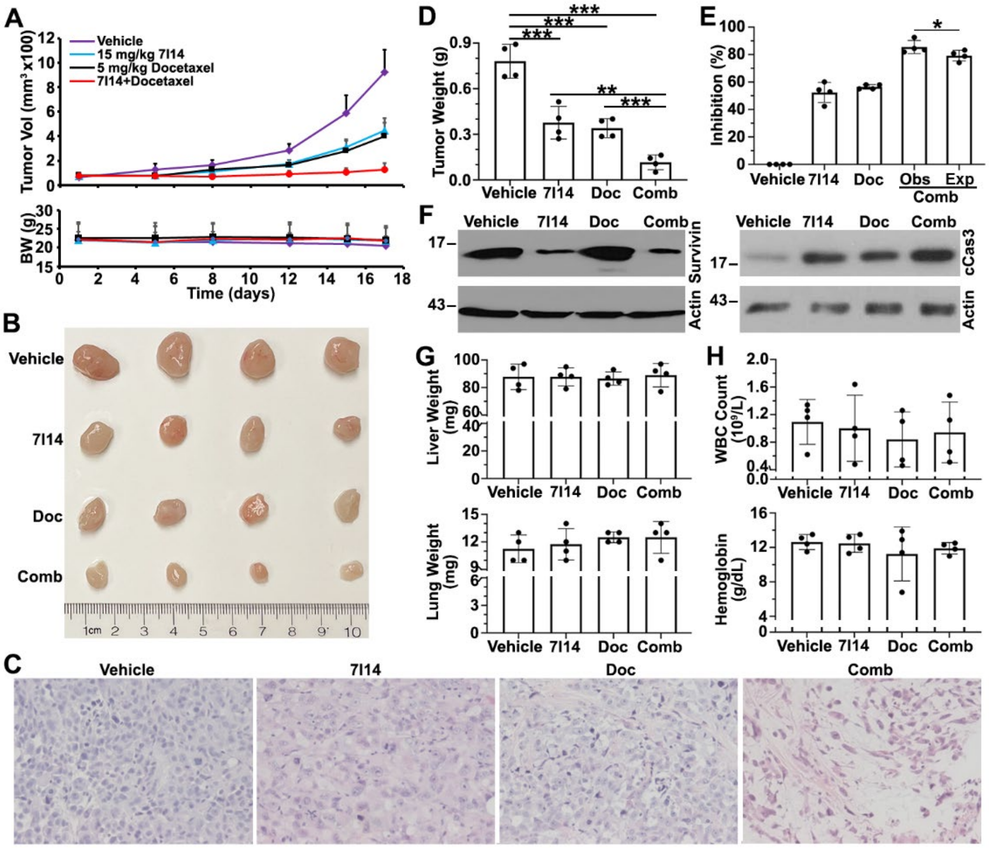

Figure S3. In-vivo activity of 7I14 and its synergism with docetaxel. (A) Effect of 7I14, docetaxel (Doc), or combination of the two agents (Comb) on the growth of PC-3 xenograft tumors and body weight (BW) of male NSG mice. **(B-D)** Gross anatomy (B), H&E staining (C), and final wet weight (D) of dissected xenograft tumors. **(E)** Tumor growth inhibition (%) by single agent or combination treatment. The expected (Exp) combination inhibition of additivity was calculated from the observed inhibition by the single agent alone using the Bliss independence model for synergy (see Materials and Methods). Obs, observed combination inhibition. **(F)** Western blot analysis of survivin, cleaved caspase 3 (cCas3), and actin loading control in the dissected xenograft tumors. Each lane represents mixture of tissue lysates from four tumors in equal proportion of the same treatment group. **(G)** Wet weight of liver and lung and (**H**) white blood cell counts and hemoglobin level in the mice of each treatment group. The data in the vehicle control and 7I14 treatment group were published previously (Cui et al., 2025) and are part of the combination study serving as controls for the combination group. The error bars represent ±SD, and one-way ANOVA were used to test statistical significance. (n=4, $^*p \leq 0.05$, and $^{**}p \leq 0.01$).
